# Variations in DNA methylation of interferon gamma and programmed death 1 in allograft rejection after kidney transplantation

**DOI:** 10.1186/s13148-016-0288-0

**Published:** 2016-11-16

**Authors:** Karin Boer, L. Elly A. de Wit, Fleur S. Peters, Dennis A. Hesselink, Leo J. Hofland, Michiel G. H. Betjes, Caspar W. N. Looman, Carla C. Baan

**Affiliations:** 1 Department of Internal Medicine, Section Nephrology and Transplantation, Erasmus MC, University Medical Center Rotterdam, Room Na520, P.O. Box 2040, 3000 CA Rotterdam, The Netherlands; 2 Department of Internal Medicine, Section Endocrinology, University Medical Center Rotterdam, Rotterdam, The Netherlands; 3 Department of Public Health, Erasmus MC, University Medical Center Rotterdam, Rotterdam, The Netherlands

**Keywords:** DNA methylation, CD8 T-cell subset, IFNγ, PD1, Kidney transplant recipients, Allograft rejection

## Abstract

**Background:**

The role of DNA methylation in the regulation of the anti-donor-directed immune response after organ transplantation is unknown. Here, we studied the methylation of two mediators of the immune response: the pro-inflammatory cytokine *interferon γ* (*IFNγ*) and the inhibitory receptor *programmed death 1* (*PD1*) in naïve and memory CD8+ T cell subsets in kidney transplant recipients receiving immunosuppressive medication. Both recipients experiencing an episode of acute allograft rejection (rejectors) as well as recipients without rejection (non-rejectors) were included.

**Results:**

CpGs in the promoter regions of both *IFNγ* and *PD1* were significantly (*p* < 0.001) higher methylated in the naïve CD8+ T cells compared to the memory T cell subsets. The methylation status of both *IFNγ* and *PD1* inversely correlated with the percentage of IFNγ or PD1-producing cells. Before transplantation, the methylation status of both *IFNγ* and *PD1* was not significantly different from healthy donors. At 3 months after transplantation, irrespective of rejection and subsequent anti-rejection therapy, the *IFNy* methylation was significantly higher in the differentiated effector memory CD45RA+ (EMRA) CD8+ T cells (*p* = 0.01) whereas the PD1 methylation was significantly higher in all memory CD8+ T cell subsets (CD27+ memory; *p* = 0.02: CD27− memory; *p* = 0.02: EMRA; *p* = 0.002). Comparing the increase in methylation in the first 3 months after transplantation between rejectors and non-rejectors demonstrated a significantly more prominent increase in the *PD1* methylation in the CD27− memory CD8+ T cells in rejectors (increase in rejectors 14%, increase in non-rejectors 1.9%, *p* = 0.04). The increase in DNA methylation in the other memory CD8+ T cells was not significantly different between rejectors and non-rejectors. At 12 months after transplantation, the methylation of both *IFNγ* and *PD1* returned to baseline levels.

**Conclusions:**

The DNA methylation of both *IFNγ* and *PD1* increases the first 3 months after transplantation in memory CD8+ T cells in kidney transplant recipients. This increase was irrespective of a rejection episode indicating that general factors of the kidney transplantation procedure, including the use of immunosuppressive medication, contribute to these variations in DNA methylation.

## Background

Kidney transplantation is currently the best treatment option for patients with irreversible, end-stage kidney disease [1]. Successful kidney transplantation is hampered by different complications including immune-mediated complications such as acute rejection [2]. Several non-invasive biomarkers for acute rejection have been studied, including proteins involved in cytotoxic lymphocyte function (e.g., perforin and granzyme B), cytokines (e.g., interferon (IFN) γ), and immune-related chemokines (e.g., CXCL9 and CXCL10) [3, 4]. Nevertheless, it remains difficult to predict and regulate the host immune response after transplantation. The host immune response is orchestrated by a tightly regulated cascade of gene expression changes which are regulated by epigenetic mechanisms like histone modifications, DNA methylation, microRNA interactions, and chromatin remodeling complexes [5–8]. Variations in these epigenetic mechanisms might serve as an additional marker to monitor the host immune response after organ transplantation.

An important player of the host immune response is the pro-inflammatory cytokine IFNγ, and high expression of IFNγ is associated with both acute and chronic allograft rejection [9–11]. The expression of *IFNγ* is regulated by DNA methylation with the addition of methyl groups on cytosine-phosphate-guanine sites (CpGs) in the *IFNγ* promoter region silencing its expression. The CpG methylation pattern of *IFNy* discriminates different T cell subsets. First, naïve (antigen unexperienced) T cells versus memory (antigen experienced) T cells (both CD4+ and CD8+ T cells) with memory T cells having a lower methylation profile [12–14]. Second, the different T helper cell (Th) subsets with Th1 cells being hypomethylated compared to the Th2 and Th17 subsets [15–17]. Another important molecule involved in the regulation of the anti-donor immune response is the inhibitory receptor programmed cell death (PD) 1. Aggressive recipient T cells that attack the transplanted organ, the so-called alloreactive T cells, are inhibited by PD1 signaling. In addition, PD1 signaling promotes the generation of induced regulatory T cells [18, 19]. The expression of *PD1* is also dependent on DNA methylation and while mainly methylated in naïve T cells, *PD1* is demethylated during differentiation into memory T cells [20].

Regulation of gene expression by DNA methylation is a well-known epigenetic mechanism with a critical role in physiological development and normal cell function by coordinating the lineage- and tissue-specific expression of genes [21]. DNA methylation is dynamic and susceptible to stimuli from the environment including internal stimuli like cytokines and hormones and external stimuli like chemical agents, pollutants, dietary components, and chronic viral infections [16, 22–24]. Aberrant DNA methylation profiles are associated with the pathogenesis of disease. Initially, DNA methylation was associated with tumor formation and progression [25], but later on, variations in DNA methylation have been associated with other diseases [26, 27] including chronic kidney disease (CKD) [28, 29] and immune-mediated diseases such as rheumatoid arthritis [30] and allergy [31, 32]. In addition, variations in DNA methylation of immune-related genes orchestrate the host immune response after organ transplantation [5–8].

Graft-infiltrating cytotoxic CD8+ T cells play a major role in the rejection process and elevated numbers of effector, and memory CD8+ T cell subsets are associated with an increased risk for acute rejection [33–35]. Here, we examined the influence of variations in DNA methylation of *IFNγ* and *PD1* in different CD8+ T cell subsets on allograft rejection. The DNA methylation of *IFNγ* and *PD1* was determined in kidney transplant recipients before and 3 and 12 months after transplantation, and both kidney transplant recipients who experienced a rejection episode within the first 3 months after transplantation and recipients who remained free from rejection were included. To exclude gender- [32] or chronic viral infection [24]-related differences, we first analyzed whether the DNA methylation of either *IFNγ* or *PD1* was different in males versus females or in cytomegalovirus (CMV) seropositive healthy donors versus CMV seronegative healthy donors.

## Results

### IFNγ methylation is significantly decreased in CMV seropositive individuals

**Fig. 1:**
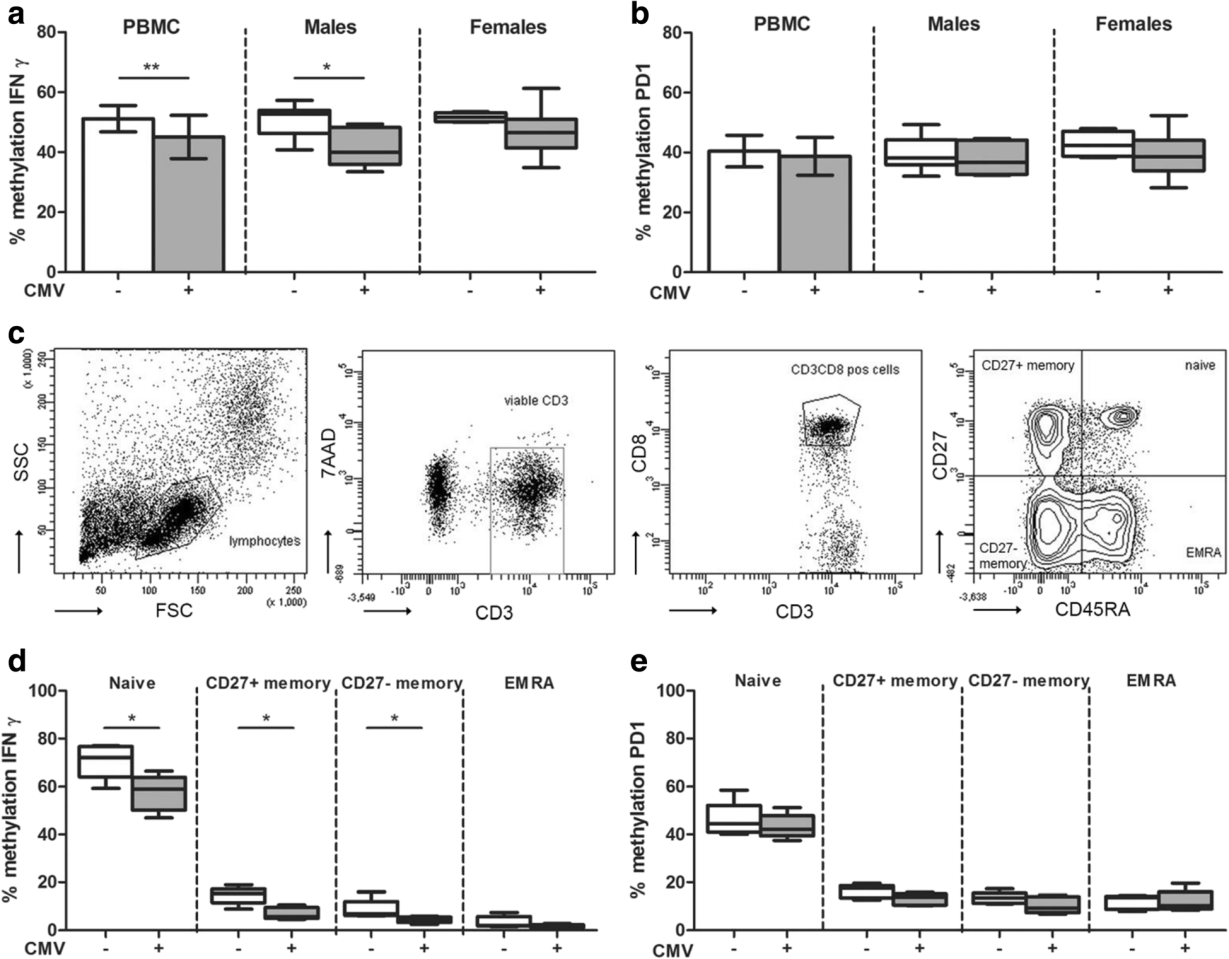
*IFNγ* and *PD1* methylation in CMV seropositive and CMV seronegative healthy kidney donors. The percentage of DNA methylation of *IFNγ* (**a**) and of *PD1* (**b**) in CMV seronegative (*n* = 15; *open bars*) and CMV seropositive healthy donors (*n* = 15; *gray bars*) in PBMCs (mean ± SD) and stratified by gender (*box and whiskers min to max*). Gating strategy of the different CD8+ memory T cell subsets in (**c**). The percentage of DNA methylation of *IFNγ* (**d**) and of *PD1* (**e**) in CMV seropositive (*n* = 5; *open bars*) and CMV seronegative healthy donors (*n* = 5; *gray bars*) in cell sorted CD8+ T cell subsets; naïve, CD27+ memory, CD27− memory, and differentiated effector memory CD45RA+ (EMRA). *Box and whiskers* (min to max); *p < 0.05 and **p < 0.01

In PBMCs of CMV seronegative healthy donors, the DNA methylation of *IFNy* was 51.2 ± 4.4% (mean ± SD). The *IFNy* methylation was significantly lower in PBMCs of age-matched CMV seropositive healthy kidney donors (45.1 ± 7.2%, *p* = 0.009; Fig. 1a). In both males and females, the methylation of *IFNy* was lower in the CMV seropositive individuals (Fig. 1a), and there was no significant difference between males and females. The DNA methylation of *PD1* in PBMCs of CMV seronegative healthy donors was comparable to the *PD1* methylation in CMV seropositive healthy donors (40.5 ± 5.3 versus 38.9 ± 6.3%; Fig. 1b). Subdividing the PBMCs into the different CD8+ T cell subsets (Fig. 1c) demonstrated significantly lower methylation of *IFNγ* in naïve, CD27+ memory, and CD27− memory CD8+ T cells in CMV seropositive individuals compared to CMV seronegative individuals (Fig. 1d). The methylation of *PD1* was not significantly different between the CMV seropositive individuals and CMV seronegative individuals in all the studied CD8+ T cell subsets (Fig. 1e).

### DNA methylation inversely correlates with protein expression

**Fig. 2:**
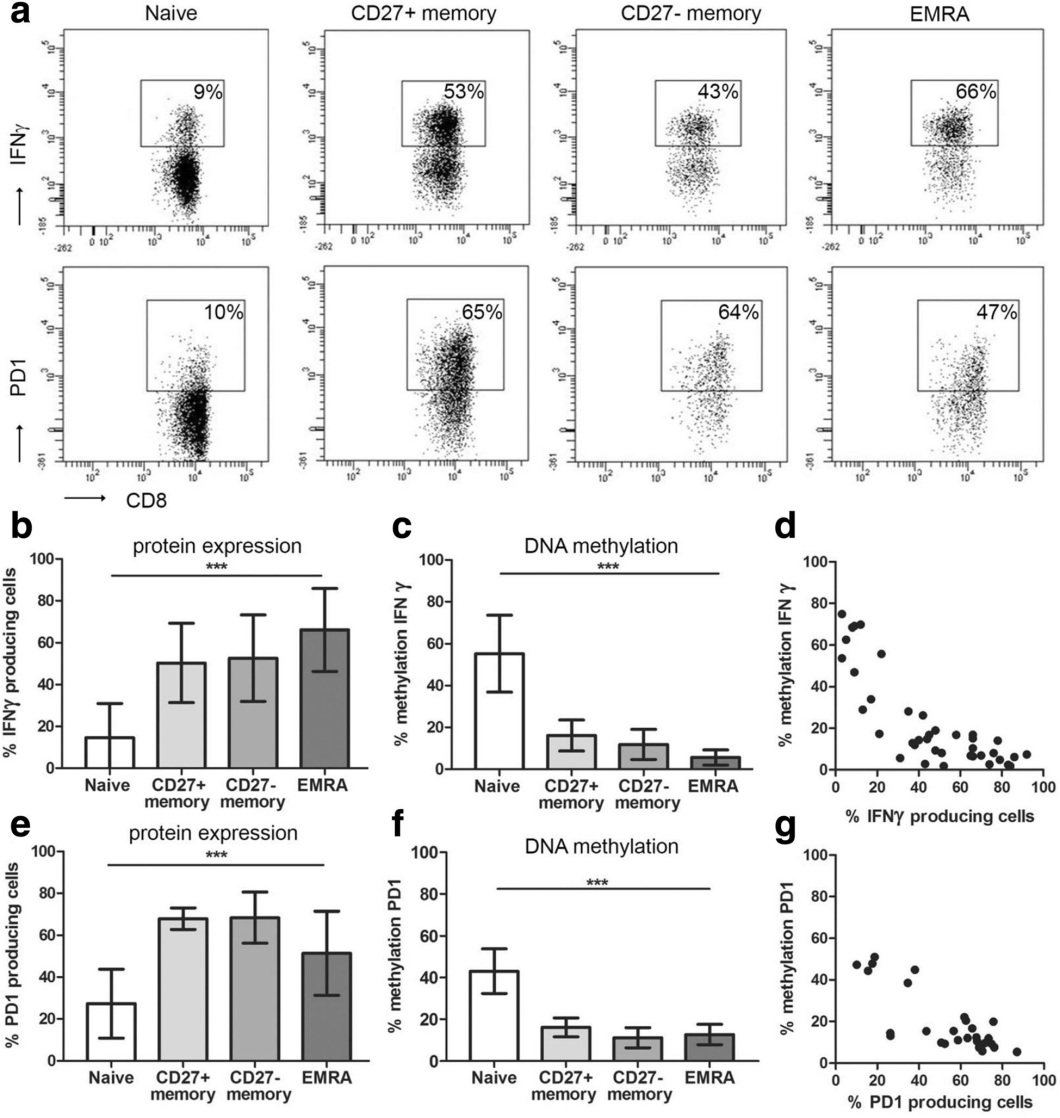
IFNγ and PD1 protein expression and *IFNγ* and *PD1* DNA methylation. FACS plots of IFNγ and PD1 expression in naïve, CD27+ memory, CD27− memory, and differentiated effector memory CD45RA+ (EMRA) CD8+ T cells in (**a**; representative example). Mean protein expression and percentage of DNA methylation in the different CD8+ T cell subsets in kidney transplant recipients before transplantation (*n* = 10; IFNγ in **b**–**d** and PD1 in **e**–**g**; mean ± SD). ***p < 0.001

To determine whether variations in DNA methylation at the described CpGs [20, 36] are associated with changes in protein expression, we measured the expression of IFNγ and PD1 in the different CD8+ T cell subsets (Fig. 2a). A clear-cut difference was observed between the naïve CD8+ T cells compared to the memory CD8+ T cells where 14.6 ± 16.4% (mean ± SD) of naïve CD8+ T cells expressed IFNγ versus 50.3 ± 18.9% of the CD27+ memory, 52.6 ± 20.6% of the CD27− memory and 66.1 ± 19.8% of the EMRA CD8+ T cells expressed IFNγ (*p* < 0.0001; Fig. 2b). In parallel, a significantly lower percentage of naïve CD8+ T cells expressed PD1 compared to the memory CD8+ T cell subsets (naïve 27.3 ± 16.5%, CD27+ memory 67.9 ± 5.1%, CD27− memory 68.4 ± 12.2%, and EMRA 51.4 ± 20.1; *p* < 0.0001; Fig. 2e). The highest percentage of IFNγ-expressing cells was found within the EMRA CD8+ T cells while the CD27+ and CD27− memory CD8+ T cell subsets contained the highest percentages of PD1-expressing cells. The DNA methylation of both *IFNγ* and *PD1* demonstrated the opposite pattern with the highest percentage of methylation in naïve CD8+ T cells. Naïve CD8+ T cells were methylated for 55.2 ± 18.3% at the *IFNγ* locus and for 43.1 ± 10.7% at the *PD1* locus. This methylation was significantly higher (*p* < 0.0001 for both *IFNγ* and *PD1*) compared to the different memory CD8+ T cell subsets (Fig. 2c, f). This inverse relation between the DNA methylation and protein expression confirms the regulatory capacity of the studied CpGs (Fig. 2d, g).

### Variations in DNA methylation in kidney transplant recipients before transplantation

**Fig. 3:**
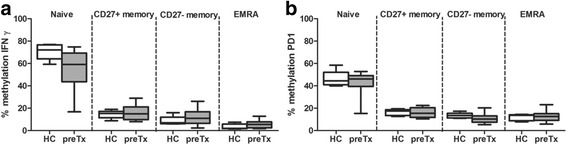
*IFNγ* and *PD1* methylation in healthy donors and kidney transplant recipients before transplantation. The percentage of DNA methylation of *IFNγ* (**a**) and *PD1* (**b**) in healthy controls (HC; *n* = 5; *open bars*) and kidney transplant recipients before transplantation (preTx; *n* = 10; *gray bars*) in cell sorted CD8+ T cell subsets; naïve, CD27+ memory, CD27− memory, and differentiated effector memory CD45RA+ (EMRA). *Box and whiskers* (min to max)

Before kidney transplantation, the methylation of *IFNγ* in CMV seronegative kidney recipients was comparable to the methylation levels in CMV seronegative healthy donors for naïve, CD27+ memory, CD27− memory, and EMRA CD8+ T cells (Fig. 3a). The same pattern was seen for the methylation of *PD1* (Fig. 3b). Subdividing the transplant recipients into the ones that went on to experience a rejection after transplantation, the rejectors and the non-rejectors, did not reveal any significant differences in methylation of *IFNγ* nor *PD1*, either between the two recipient groups or in comparison to the healthy donors (data not shown).

### Variations in DNA methylation in kidney transplant recipients after transplantation

**Fig. 4:**
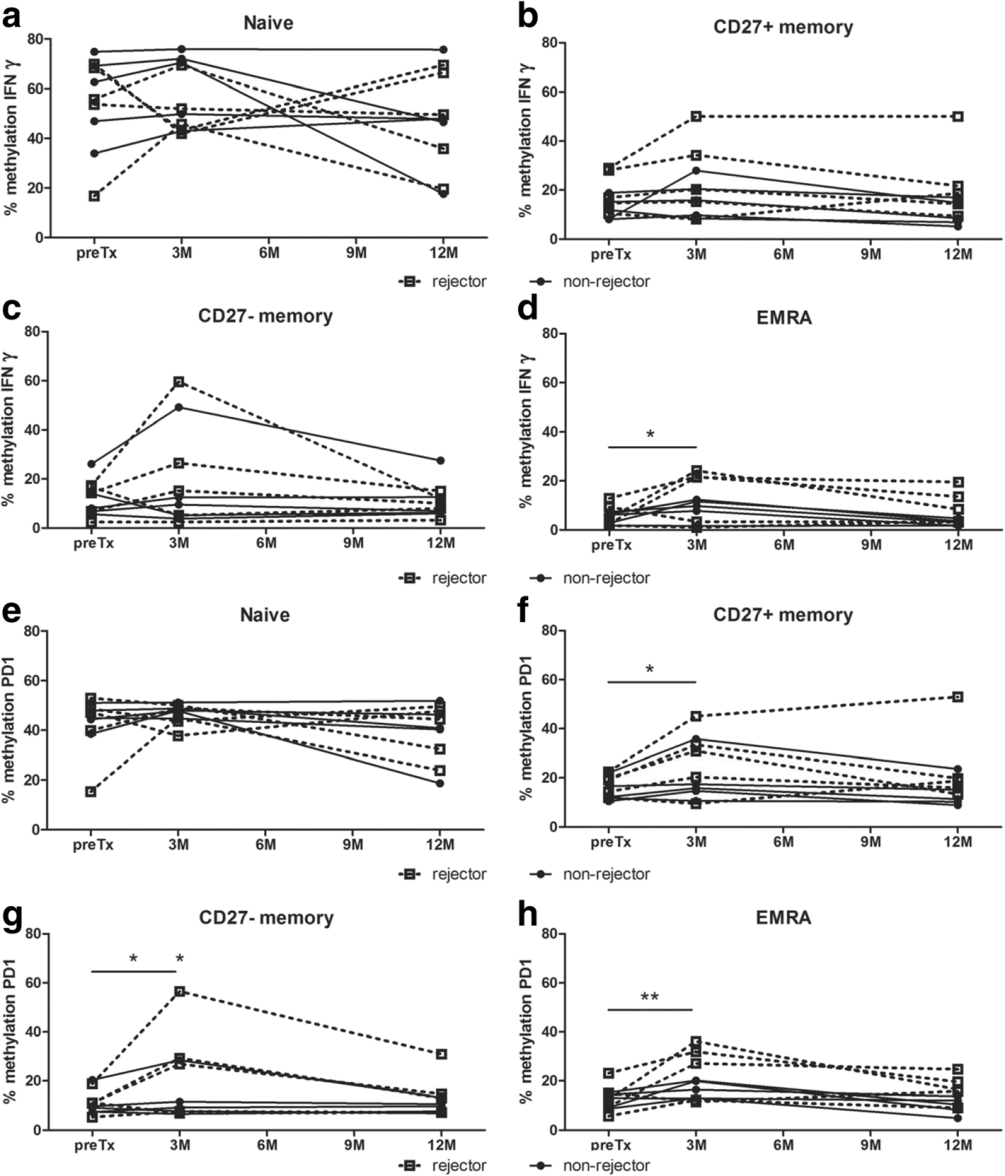
*IFNγ* and *PD1* methylation in kidney transplant recipients during the first year after transplantation. The percentage of DNA methylation of *IFNγ* (**a**–**d**) and of *PD1* (**e**–**h**) in kidney transplant recipients before and 3 and 12 months after transplantation in cell sorted CD8+ T cell subsets; naïve (**a**, **e**), CD27+ memory (**b**, **f**), CD27− memory (**c**, **g**), and differentiated effector memory CD45RA+ (EMRA; **d**, **h**). *p < 0.05 and **p < 0.01

After kidney transplantation, the percentage of methylation of *IFNγ* did not change significantly in the naïve, CD27+ memory, and CD27− memory CD8+ T cells during the first year after transplantation (Fig. 4a–c). In the EMRA CD8+ T cells, the methylation of *IFNγ* was significantly higher at 3 months after transplantation compared to the methylation before transplantation irrespective of rejection and the subsequent anti-rejection therapy (*p* = 0.01; Fig. 4d). Focusing on rejection demonstrated that the methylation of *IFNγ* was significantly higher at 3 months after transplantation in the rejectors (14.3% versus 6.3% before transplantation; *p* = 0.01) while the non-rejectors increased from 4.9 to 8.6% (not significant). Both rejectors and non-rejectors demonstrated elevated *IFNγ* methylation levels in the EMRA CD8+ T cells at 3 months after transplantation, but this increase in methylation was not significantly different between rejectors and non-rejectors (*p* = 0.3). At 1 year after transplantation, the methylation of *IFNγ* was comparable to the levels measured before transplantation.

The methylation of *PD1* did not change significantly in the naïve CD8+ T cells during the first year after transplantation (Fig. 4e). Irrespective of rejection, the methylation of *PD1* significantly increased during the first 3 months after transplantation in CD27+ memory CD8+ T cells with 7.2% (*p* = 0.02), in CD27− memory CD8+ T cells with 7.9% (*p* = 0.02), and in EMRA CD8+ T cells with 7.5% (*p* = 0.002; Fig. 4f–h)). Focusing on rejection demonstrated a more prominent increase in DNA methylation in the rejectors compared to the non-rejectors in all memory CD8+ T cell subsets (CD27+ memory: rejectors 27.8 versus 17.6%, *p* = 0.02 and non-rejectors 18.9 versus 14.6% *p* = 0.3; CD27− memory: rejectors 25.4 versus 11.4%, *p* = 0.002 and non-rejectors 12.7 versus 10.9%, *p* = 0.6; EMRA: rejectors 23.8 versus 13.2%, *p* = 0.002 and non-rejectors 16.5 versus 12.1%, *p* = 0.2; methylation at 3 months versus before transplantation, respectively). The increase in *PD1* methylation in rejectors during the first 3 months after transplantation was not significantly different from the increase in *PD1* methylation in non-rejectors in both the CD27+ memory CD8+ T cells (*p* = 0.3) and EMRA CD8+ T cells (*p* = 0.2). In the CD27− memory CD8+ T cells, the increase in *PD1* methylation was significantly higher in the rejectors (14%) compared to the non-rejectors (1.9%, *p* = 0.04). In parallel with the methylation of *IFNγ*, the methylation of *PD1* returned to normal levels at 1 year after transplantation.

## Discussion

The clinical potential of DNA methylation in organ transplantation, either as diagnostic or prognostic biomarker or as therapeutic target has been proposed by many [5–8, 37, 38]. Nevertheless, this is the first study where DNA methylation of two selected genes, *IFNγ* and *PD1*, was actually studied in CD8+ T cells in a small cohort of human kidney transplant recipients over time in relation to acute allograft rejection. Irrespective of rejection, we observed at 3 months after transplantation significantly elevated DNA methylation levels of *IFNγ* in the differentiated EMRA CD8+ T cells, while the DNA methylation of PD1 was significantly higher in all CD8+ memory T cell subsets. This increase in *IFNγ* methylation was not significantly different between rejectors and non-rejectors, while the increase in *PD1* methylation was significantly higher in the rejectors in the CD27− memory CD8+ T cells. In the other CD8+ memory T cells subsets (CD27+ memory and EMRA), the increase in DNA methylation of *PD1* was not significantly different between rejectors and non-rejectors.

Kidney transplantation will activate the recipient’s immune system accompanied by an increase in cytokine production, including production of the pro-inflammatory IFNγ [35, 39, 40], and upregulation of PD1 expression [41]. As protein expression inversely correlates with DNA methylation levels at gene promoter sites, kidney transplantation induces demethylation of genes involved in immune activation. However, for both *IFNγ* and *PD1*, an increase in DNA methylation was observed in rejectors and non-rejectors in the first 3 months after transplantation, indicative for lower expression levels of IFNγ and PD1. Likely, the expected demethylation is only detectable in the donor-antigen specific T cells. The low percentage of these cells within the selected CD8+ T cells explains why the expected decrease in methylation was not observed. The observed increase in *IFNγ* and *PD1* DNA methylation most likely does not reflect the immune response against the foreign donor antigen but demonstrates a downregulation of the immune system achieved by the given immunosuppressive medication which non-specifically block all T cell subsets. For example, the usage of prednisolone. In this study, prednisolone was tapered to 5 mg at month 3 and thereafter completely withdrawn. At 1 year after transplantation, the DNA methylation levels returned to baseline.

In a clinical transplantation setting, it is impossible to measure the DNA methylation of either *IFNγ* or *PD1* just before rejection. Currently, rejection cannot be predicted as the moment of rejection strongly varies between individuals and therefore those samples are not available. Although the material was only available of a small number of patients, we had the unique opportunity to follow the same patients over time. Variations in DNA methylation are more profoundly found in the period after withdrawal of stress exposure (e.g., drugs) compared to the period during exposure [42, 43]. Translation to the field of organ transplantation implies that after a rejection episode including anti-rejection therapy, rejectors would have more variations in DNA methylation compared to non-rejectors. However, this was not true for the methylation of either *IFNγ* or *PD1* at 12 months after transplantation, indicating that allograft rejection has no imprinted effect on the DNA methylation of those immune genes.

Despite differences in immune activity of the distinct memory CD8+ T cell subsets, the variations in DNA methylation in either memory subset were comparable. The EMRA CD8+ T cells are potentially the most aggressive subtype with a strong cytolytic activity, while the CD27+ memory cells display weak cytolytic activity producing effector cytokines such as interleukin (IL) 2, IFNγ, tumor necrosis factor (TNF) α, and IL4 [44, 45]. The CD27− memory CD8+ T cells, which are functionally in between the CD27+ memory CD8+ T cells and the EMRA CD8+ T cells, represent the smallest subpopulation and it is unclear why specifically these cells demonstrated a significant difference in increase in methylation of *PD1* between rejectors and non-rejectors.

DNA methylation is adjustable by cues from the environment, e.g., viral infections [20, 24, 46], though the exact cues and mechanisms remain largely unknown [16, 22, 23]. The uremic condition during chronic kidney disease (CKD) modifies DNA methylation profiles [47–49]. Although, before transplantation, we did not observe significant changes in the methylation of either *PD1* or *IFNγ* compared to age-matched healthy donors. Either the previously observed effect on DNA methylation is gene specific and not applicable to *IFNy* and *PD1* or the included transplant recipients here had less severe kidney disease compared to the CKD patients studied previously.

In contrast to previous observations where males demonstrated a significantly higher DNA methylation of *IFNγ* compared to females [32], significant differences in DNA methylation between males and females were not observed. However, we observed a significantly lower % of *IFNγ* methylation in CMV seropositive healthy donors compared to CMV seronegative healthy donors. The effect of chronic CMV infection on DNA methylation is not documented yet, but the change of the composition of the T cell pool with a permanent increase in highly differentiated T cells with a more memory phenotype in CMV seropositive individuals [50] has been demonstrated repeatedly. Therefore, the lower % of *IFNγ* methylation in CMV seropositive individuals might be explained by the fact that memory T cells are less methylated at the *IFNγ* locus (Fig. 2 and [12–14]). Nevertheless, also in selected CD8+ memory T cells, the methylation of *IFNγ* was significantly lower in the CMV seropositive individuals (Fig. 1), indicating that CMV infection not only affects the composition of the T cell compartment but also induces a more aggressive T cell phenotype since demethylation is associated with an increased IFNγ production.

Although we could not identify variations in DNA methylation of either *IFNy* or *PD1* in CD8+ T cells which could either diagnose or predict allograft rejection after kidney transplantation, further research is needed to appreciate the clinical significance of variations in DNA methylation and other epigenetic mechanisms in kidney transplantation. Epigenetic biomarkers, mainly based on variations in DNA methylation, are well established in the diagnosis of cancer and are not only detectable in the affected tissue as well as in the urine or the peripheral blood [51, 52]. Currently, the application of epigenetic biomarkers is extended to other complex diseases such as autoimmune diseases [30, 53, 54]. The increasing knowledge on the epigenetic regulation of immune cells will contribute to our understanding of the epigenetic regulation of the complex anti-donor immune response after kidney transplantation. Epigenetic variations precede changes in protein expression and cell function and thereby represent an early indicator of clinical complications. Accordingly, a more comprehensive understanding of the epigenetic regulation of the anti-donor immune response will learn whether variations in DNA methylation can serve as predictive, diagnostic, or prognostic markers. Moreover, since DNA methylation is influenced by environmental cues, it might serve as a target for therapeutic intervention.

A genome-wide approach instead of selected immunoregulatory genes are a good option for future research. Genome-wide analysis enables the identification of variations in DNA methylation in all promoter regions as well as other gene regions including intragenic and intergenic regions [47, 55, 56]. Since DNA methylation profiles are cell-type specific [57], selected cell subsets involved in the anti-donor immune response (e.g., CD4+ T cell subsets, B cells, and macrophages), or even better the donor-antigen specific cells, should be analyzed. Another interesting, though technically more challenging option, is to analyze variations in DNA methylation in graft-infiltrating T cells. As variations in DNA methylation occur specifically in donor-antigen specific cells which are more abundantly present in the graft compared to the circulation.

## Conclusions

After kidney transplantation, the DNA methylation of the promoter of both *IFNγ* and *PD1* increases in the first 3 months and returns to baseline at 1 year after transplantation irrespective of rejection. These variations do not reflect the anti-donor immune response but are more likely the result of the transplantation procedure and the use of immunosuppressive medication.

## Methods

### Study population

Prior to the selection of kidney transplant recipients, we first determined whether cytomegalovirus (CMV) infection modulates DNA methylation of either *IFNγ* or *PD1*. Peripheral blood mononuclear cells (PBMCs) of 15 CMV seropositive healthy donors (age 52 years, range 38–71; 5 males and 10 females) and 15 age-matched CMV seronegative healthy donors (age 52 years, range 44–59; 11 males and 4 females) were studied. Of these 30 healthy donors in total, we selected 5 CMV seropositive and 5 CMV seronegative age-matched individuals to study the methylation status in different CD8+ T cell subsets. Based on the significant decrease in DNA methylation of *IFNγ* in CMV seropositive healthy donors, we included only CMV seronegative kidney transplant recipients who received their first kidney from a living donor. The DNA methylation of both *IFNγ* and *PD1* was examined in different CD8+ T cell subsets in 5 recipients who developed a biopsy-proven acute cellular rejection within the first 3 months after transplantation (rejectors; Table 1) and 5 age-matched recipients who remained free from rejection the first year after transplantation (non-rejectors) and was compared to 5 age-matched healthy donors (age 54 years, range 44–59). The different CD8+ T cell subsets were analyzed at different time points; before transplantation and 3 and 12 months after transplantation. The selected CMV seronegative recipients all received a kidney from a CMV seronegative donor and received basiliximab as induction therapy. After transplantation, recipients received standard triple maintenance therapy consisting of prednisolone (tapered after 3 months), mycophenalate mofetil (MMF), and tacrolimus. Anti-rejection therapy consisted of methylprednisolone (1 g per day) on three consecutive days followed in some cases by anti-thymocyte globulin (ATG; *n* = 2) or alemtuzumab (*n* = 1).

**Table 1:** Clinical characteristics of kidney transplant recipients

	Rejectors	Non-rejectors
No. of subjects	5	5
Age at transplantation (year)^a^	47 (43–54)	52 (44–66)
Gender (M/F)	4/1	5/0
Serum creatinin (μmol/l)^a,b^	480 (270–1484)	532 (374–682)
Underlying kidney disease		
HN/PKD/other	3/1/1	0/4/1
Renal replacement therapy		
HD/PD/pre-emptive	1/2/2	1/1/3
Number of HLA-A/B mismatches^c^	2.2 ± 0.4	2.8 ± 0.8
Number of HLA-DR mismatches^c^	2.0 ± 0	1.0 ± 0.7

*HN* hypertensive nephropathy, *PKD* polycystic kidney disease, *HD* hemodialysis, *PD* peritoneal dialysis

^a^Median with range

^b^Before transplantation

^c^Mean ± SD

### Isolation of peripheral blood mononuclear cells and CD8+ T cell subsets

Peripheral blood mononuclear cells (PBMCs) were isolated from heparinized blood samples by density gradient centrifugation using standard Ficoll-Paque (GE Healthcare, Uppsala, Sweden) procedures. Since DNA methylation profiles are cell type specific [57], we examined naïve (antigen unexperienced; CD27+CD45RA+) CD8+ T cells and memory (antigen experienced) CD8+ T cells separately. The memory CD8+ T cells were subdivided into the differentiated effector memory CD8+ T cells (EMRA: CD27-CD45RA+, with a strong cytolytic activity), CD27+ memory T cells (CD27+CD45RA−; with weak cytolytic potential), and CD27− memory T cells (CD27-CD45RA−; functionally in between CD27+ memory CD8+ T cells and EMRA CD8+ T cells) [44, 45]. The different CD8+ T cell subsets were isolated using cell sorting (BD FACSAria™ II SORP, BD Biosciences, San Jose, CA, USA) with a mean purity of 96%. Total PBMCs were stained with the following monoclonal antibodies: Brilliant Violet 510™-labeled CD3 (Biolegend, San Diego, CA, USA), APC-Cy7-labeled CD8 (BD), PE-Cy7-labeled CD27 (eBioscience, San Diego), APC-labeled CD45RA (BD), and 7-amino-actinomycin D (7-AAD, BD) for the exclusion of nonviable cells.

### Bisulfite conversion

PBMCs and the FACS-sorted CD8+ T cell subsets were digested with proteinase K and treated with bisulfite using the EZ DNA Methylation-Direct Kit (Zymo Research from Base Clear Lab products, Leiden, The Netherlands), according to the manufacturer’s instructions. During bisulfite treatment, unmethylated cytosines were converted into uracil, whereas methylated cytosines remained unchanged.

### PCR amplification and pyrosequencing

The DNA methylation of the *IFNγ* promoter was determined at two CpGs (CpG-186 and CpG-54) with transcription factor activity [36], and for *PD1*, eight previously described [20] CpG sites ranging between −914 and −738 bp from the start codon were studied (CpG-914, CpG-911, CpG-906, CpG-857, CpG-833, CpG-776, CpG-762, and CpG-738). Since the methylation status at adjacent CpGs is correlated [58], the mean % of methylation of either *IFNγ* or *PD1* was calculated. Primers for PCR and pyrosequencing were designed using PyroMark Assay Design 2.0 software (Qiagen, Venlo, The Netherlands; Table 2).

**Table 2:** Primers for PCR amplification and pyrosequencing

Gene	Primers	CpGs
*IFNγ*	F: 5′-ATGGTATAGGTGGGTATAATGG-3′	
	R: 5′-biotin-CAATATACTACACCTCCTCTAACTAC-3′	
	S: 5′-GGTGGGTATAATGGG-3′	CpG-186
	S: 5′-ATTATTTTATTTTAAAAAATTTGTG-3′	CpG-54
*PD1*	F: 5′-AGTATAGAATATAAGGAGATAAGTAAGT-3′	
	R: 5′-biotin-CCATAACCACAATTCCAAATCTTT-3′	
	S: 5′-AGAATATAAGGAGATAAGTAAGTT’-3′	CpG-914, CpG-911, CpG-906
	S: 5′-GGATTTTTTGAATTATTTTATTTTG′-3′	CpG-857, CpG-833
	S: 5′-TTAGTTTTATAGTTAGTTTTTG-3′	CpG-776, CpG-762, CpG-738

*F* forward primer, *R* reverse primer, *S* sequencing primer, *CpGs* cytosine phosphate guanine sites

PCR amplifications were performed with the Pyromark PCR Kit from Qiagen with each primer in a concentration of 0.2 μM. The PCR conditions were 15 min at 95 °C, 45 cycles of 30 s 94 °C, 30 s 58 °C for *IFNγ* and 56 °C for *PD1* and 30 s 72 °C followed by 10 min at 72 °C and on hold at 21 °C. After visualization of the appropriately sized PCR product on a 1% agarose gel, the PCR product was sequenced using a PyroMark Q24 pyrosequencer (Qiagen) with the following minor revisions to the manufacturer’s instructions: to immobilize the PCR product, 1 μl Streptavidin Sepharose High Performance Beads (GE Healtcare) were used per sequence reaction and annealing of the sequence primers was done for 3 min at 80 °C. The bisulfite conversion and the subsequent PCR amplification and pyrosequencing were performed in duplicate. Human low and high methylated DNA from EpigenDx (Hopkinton, MA, USA) were used as controls.

### IFNγ and PD1 protein expression

To determine IFNγ and PD1 protein production by the different CD8+ T cell subsets, total PBMCs were either not stimulated or stimulated in the presence of 1 μg/ml Brefeldin A (GolgiPlug; BD Biosciences) with PMA (50 ng/ml, Sigma-Aldrich, St. Louis, MO, USA) and ionomycin (1 μg/ml, Sigma-Aldrich) for 4 h at 37 °C in 5% CO_2_. For IFNγ, cells were stained for 30 min for the following surface markers: Brilliant Violet 510™-labeled CD3 (Biolegend), APC-Cy7-labeled CD8 (BD Biosciences), PE-Cy7-labeled CD27 (eBioscience), APC-labeled CD45RA (BD Biosciences), and 7-amino-actinomycin D (7-AAD, BD Biosciences), fixed, permeabilized, and stained with FITC-labeled IFNγ (BD Biosciences) for 30 min. Frequencies of IFNγ-producing CD8+ T cell subsets were corrected for background determined with the unstimulated condition. For PD1, cells were stained with the previously described surface markers while PE-labeled PD1 (Biolegend) was added. For PD1 expression, a Fluorescence Minus One (FMO) was used to correct for background staining. Samples were measured on the FACSCanto II (BD) and analyzed using FACSDiva software version 6.1.2. (BD).

### Statistical analysis

To identify differences between groups, the unpaired *t* test, Mann-Whitney *U* test, and ANOVA were used as appropriate. To determine differences after kidney transplantation over time between rejectors and non-rejectors, we used multilevel analysis with the percentage of DNA methylation as outcome. Predictors were different individuals (rejectors and non-rejectors), time also as categorical predictor (levels 0 (before transplantation), 3 and 12 months after transplantation), and individuals as random intercept. Each model was applied for the four different cell types studied; naïve, CD27+ memory, CD27− memory, and EMRA CD8+ T cell subsets. Afterwards, we added models with interaction between type of individual and time. The first model describes the same pattern over time for both rejectors and non-rejectors while the second one enables to estimate and test different trends in time for rejectors and non-rejectors. The estimates and standard errors were transformed to CI’s and *p* values. We used the package R version 3.1.2 and libraries lmer and lmerTest. A *p* value of <0.05 was considered statistically significant.

## Abbreviations

CKD: Chronic kidney disease
CMV: Cytomegalovirus
CpGs: Cytosine phosphate guanine sites
FMO: Fluorescence Minus One
IFNγ: Interferon γ
IL: Interleukin
PBMCs: Peripheral blood mononuclear cells
PD1: Programmed death 1
Th: T helper cell
TNFα: Tumor necrosis factor α
